# Different Socio-Demographic and Lifestyle Factors Can Determine the Dietary Supplement Use in Children and Adolescents in Central-Eastern Poland

**DOI:** 10.3390/nu11030658

**Published:** 2019-03-18

**Authors:** Ewa Sicińska, Barbara Pietruszka, Olga Januszko, Joanna Kałuża

**Affiliations:** Department of Human Nutrition, Faculty of Human Nutrition and Consumer Sciences, Warsaw University of Life Sciences (WULS—SGGW), 159c Nowoursynowska str., 02-776 Warsaw, Poland; barbara_pietruszka@sggw.pl (B.P.); olga_januszko@sggw.pl (O.J.); joanna_kaluza@sggw.pl (J.K.)

**Keywords:** adolescents, children, determinants, dietary supplements, food choice

## Abstract

Vitamin/mineral supplement (VMS) use has become increasingly popular in children and adolescents; however, different predictors may be associated with their usage. Therefore, the aim of this study was to compare determinants of VMS use in 1578 children and adolescents. Data was collected among parents of children (≤12 years old) and among adolescents (>12 years old) who attended public schools by a self-administered questionnaire. Multivariate-adjusted logistic regression models were used to estimate odds ratios (ORs) and 95% confidence intervals (95% CIs) for determining the predictors of VMS use. In children, the following determinants of VMS use were indicated: socioeconomic status (average vs. very good/good; OR: 1.69, 95% CI: 1.16–2.48), physical activity (1–5 vs. <1 h/week; OR: 1.44, 95% CI: 1.02–2.04), BMI (≥25 vs. 18.5–24.9 kg/m^2^; OR: 0.67, 95% CI: 0.46–0.98), and presence of chronic diseases (yes vs. no; OR: 2.32, 95% CI: 1.46–3.69). In adolescents, gender (male vs. female; OR: 0.56, 95% CI: 0.37–0.87), residential area (rural vs. urban; OR: 0.63, 95% CI: 0.40–0.99), BMI (<18.5 vs. 18.5–24.9 kg/m^2^; OR: 0.35, 95% CI: 0.17–0.73), and health status (average/poor vs. at least good; OR: 1.96, 95% CI: 1.13–3.39) were factors of VMS use. In both groups, the mother’s higher educational level, fortified food consumption and diet modification towards better food choices were predictors of VMS use. In conclusion, most of the predictors of VMS use were different in children and adolescents.

## 1. Introduction

Dietary supplement use, especially containing vitamin/mineral supplements (VMSs), has become increasingly popular in developed countries. About half of the US adult population [1] and more than 30% of children and adolescences (<18 years old) [2] are users of these products. In Europe, depending on the country and gender, supplement use ranges from 2% to 66% in adults [3], and from 16% to 45% in children and adolescents in England, Scotland, Germany, Slovenia, and Finland [4,5,6]. Evidence of the benefits of using VMSs among those who eat properly are insufficient and conflicting [7]; studies have shown that the use of VMSs is usually associated with better dietary habits; however, knowledge on using supplements by adults as well as children and adolescents is insufficient [8,9]. Socio-demographic and lifestyle factors like age, sex, income, education level, health status, or physical activity may associate with dietary supplement usage [8]. Surveys on the determinants of VMS use focus mostly on adults [8], while the number of studies conducted in children (aged 2–10) [10,11] and adolescents (aged 11–19) [5,12,13] is limited, and often combine both age groups together [2,14,15,16,17,18]. Moreover, most of them were carried out in the US population [2,11,12,13,14,15,16]. Taking into the consideration that, in the case of children, parents decide whether to give a child a dietary supplement, while adolescents often make this decision by themselves based on advertisements, peers’ or coach’ recommendation [6], factors which determine the usage of dietary supplements may be different in both groups. The aim of the study was to compare socio-demographic and lifestyle determinants of VMS use in school children and adolescents in Central-Eastern Poland.

## 2. Materials and Methods

### 2.1. Study Design

The cross-sectional study was conducted in school students who attended public primary and secondary schools. The schools were selected randomly in Central-Eastern Poland based on a list of public schools in this area. The survey was conducted only in those schools of which headmasters gave consent to it. The following inclusion criteria of the students participating in the study were used: attendance to a public primary or secondary school in Central-Eastern Poland and the age of participants from 5–20 years. The exclusion criteria constituted: occurrence of a disease requiring a special diet, pregnancy or lactation (it concerned adolescents only) and incorrectly or incompletely filling in a health and lifestyle questionnaire.

The survey was conducted in accordance with the guidelines laid down by the Declaration of Helsinki. The study design and protocol were approved by the Department of Human Nutrition, Warsaw University of Life Sciences (WULS-SGGW), Poland.

### 2.2. Study Population and Data Collection

**Figure 1:**
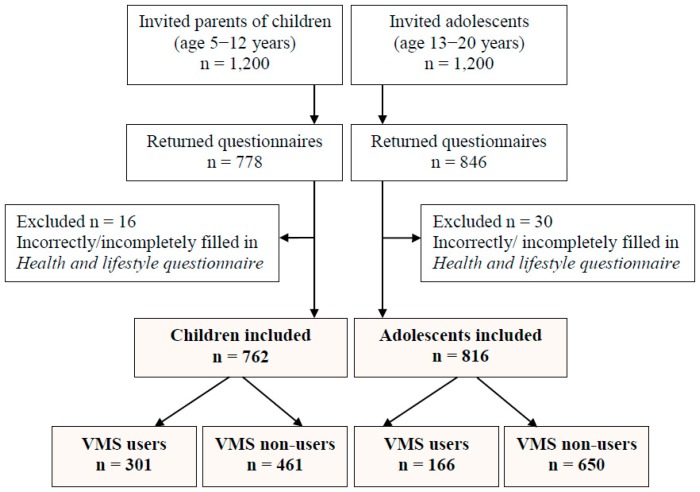
Flow chart of the study population. VMS: vitamin/mineral supplements. Notes: cream colour indicates study groups.

For the study 1200 parents of children (age 5–12 years) and 1200 adolescents (age 13–20 years) were invited to participate (Figure 1). The data was collected using the health and lifestyle questionnaire, which was distributed to parents at school meetings and to adolescents during classroom sessions. Completed questionnaires were verified by trained interviewers. A total of 1624 respondents returned the questionnaires (67.7%); however, due to incorrectly or incompletely filled in questionnaires, 46 were excluded. Finally, 1578 school students were included in the study. By completing the questionnaire, the respondents agreed to participate in the study.

The questionnaire contained 27 questions and was composed of the following sections: socio-demographic characteristics (age, gender, place of living, mother’s educational level, economic status of family); health and lifestyle status (health status, occurrence of chronic diseases, use and type of special diet, type and time spent on being physically active); eating habits (number of meals/day consumed habitually, regularity of main meal consumption, diet modification); dietary supplement usage; and voluntarily fortified foods usage. Self-reported height and weight of participants were collected and used to calculate the Body Mass Index (BMI) [19,20]. Details of the study methods have been enumerated elsewhere [21]. The questionnaire was developed and verified in an earlier study among students at the Department of Human Nutrition, WULS-SGGW, Poland [22,23].

### 2.3. Assessment of VMS Use

The respondents were asked about VMS taken during the year before the study as well as on the test day, about the name, and brand of dietary supplements, the usage form (i.e., pills, powder, drops etc.), and the period of application. As supplement users were considered those participants who used one or more VMSs over the 12 months before the survey. The study included short-term VMS users (from at least seven consecutive days to less than one month), medium-term users (1–3 months) and long-term users (>3 months). VMSs were categorised into: single vitamin; single mineral; multivitamin and/or mineral(s); and multicomponent supplement containing vitamin(s) and/or mineral(s) plus other ingredients including herbs or other.

When the respondents have declared VMS use, they were asked to give the reason for including these products in the diet by marking one or more predefined reasons: “Respondent’s diet is poor in nutrients”, “VMSs improve overall health”, “VMSs are necessary when medicines are used”, “VMSs were recommended by a physician”, as well as they could list their own reason. When the respondents have declared not using VMSs, they were asked about the reason by marking one or more of the following statements: “Lack of effect on health improvement”, “No need to use such products because of proper nutrition”, “The use of VMSs can be harmful”, “VMSs are too expensive”, as well as they could list their own reason.

### 2.4. Statistical Analysis

All results were presented separately for children (≤12 years) and adolescents (>12 years). The statistically significant differences between categorical variables and VMS usage were determined using the Chi-square test. To examine the associations between VMS usage and parameters that might constitute the determinants of VMS use, the univariate (crude data) as well as multivariate-adjusted odds ratios (ORs) with 95% confidence intervals (95% CIs) were calculated using logistic regression models. The Hosmer-Lemeshow criterion was used to evaluate the models’ goodness-of-fit.

The multivariate-adjusted models included potential determinants of VMS usage, i.e., age (continuous variable, years), gender (female or male), residential area (urban or rural), mother’s education level (primary, high school, or university), socioeconomic status (very good/good, average, or poor), time spent on being physically active (<1, 1–5, or ≥6 h/week; not including gymnastic classes in school), BMI (<18.5, 18.5–24.9, or ≥25 kg/m^2^), self-reported health status (at least good, or average or poor), presence of chronic diseases (no or yes), using a special diet (no or yes), number of meals consumed per day (≤3, 4, or ≥5), consumption of fortified foods (no or yes), and intentional diet modification towards better food choices (lack of modification, excluding or including some food products, or simultaneously excluding and including some food products). Supplement non-users were the referent category for the logistic regression models. Missing data on socio-demographic and lifestyle determinants were modelled as separate categories. The statistical analyses were performed using SPSS version 23.0, and *p*-values ≤ 0.05 were considered statistically significant.

## 3. Results

### 3.1. Characteristics of the Study Population

Over the past 12 months, 29.6% of participants used VMSs, while 15.1% used VMSs on the test day. A statistically significantly higher number of children (mean age 8.6 ± 1.4 years) compared to adolescents (mean age 17.1 ± 2.1 years) used these products during the last year (39.5% vs. 20.3%, respectively; *p*-value < 0.001). In both groups, significantly more mothers of VMS users compared to non-users had a university education (Table S1). Compared to non-users, children classified as supplement users spent more time on being physically active and suffered from chronic diseases; the same tendencies were observed in adolescents. In both groups, VMS users vs. non-users declared the consumption of fortified foods more frequently, as well as modified their diet towards better food choices by intentionally including (e.g., vegetables, fruits) or/and excluding (e.g., sweets, crisps) some foods. It was observed that significantly more children who were VMS non-users than users were overweight or obese (BMI ≥ 25 kg/m^2^; 33.1% vs. 23.6%, respectively); in contrast, in adolescents, more VMS non-users than users were underweight (BMI < 18.5 kg/m^2^; 14.4% vs. 6.0%, respectively). A description of selected factors associated with the dietary supplement use by a part of the younger respondents, i.e., group of children aged 6–12, was also presented elsewhere [24].

### 3.2. Types and Duration of VMS Use and Reason for Using/Non-Using

In both groups, the most frequently used type of VMSs were multicomponent supplements containing multivitamin and/or mineral supplements with or without other ingredients, while single vitamin and single mineral were used less commonly (Table 1). As the main reasons for VMS use, parents of children and adolescents pointed out improving overall health, diet poor in nutrients, and physician recommendations. VMS non-users indicated that they did not need them because of proper nutrition. Moreover, parents of children more often than adolescents indicated as a reason not to use VMS a lack of effect on health improvement, harmful impact on health and too high of a price.

The distribution of children and adolescents using specific nutrients with vitamin/mineral supplements (VMSs) stratified by duration of use are presented in Table 2. Compared to adolescents, the number of children using vitamin supplements (A, E, D, C, B_1_, B_2_, niacin, B_6_, folic acid, B_12_, biotin, and pantothenic acid) was statistically significantly higher, whereas magnesium and iron supplements were consumed by a significantly lower number of children. Amongst children and adolescents, the largest number of respondents used vitamin C supplements (83.7% and 65.7%, respectively). The majority of respondents were classified as medium-term VMS users (45.8%), followed by short-term users (31.5%) and lastly long-term users (22.7%). Generally, more adolescents than children were classified as medium-term users of specific nutrients with VMSs, while children respondents were more often classified as short-term and long-term users with statistically significant differences found for vitamins C, B_1_, B_2_, niacin, B_6_, B_12_, and zinc.

### 3.3. Determinants of VMS Use in Children and Adolescents

Multivariate-adjusted odds ratios (ORs) of VMS use by socio-demographic and lifestyle factors in children and adolescents are presented in Table 3. There was found an association between mother’s education level and usage of supplements in children. Compared to children of mothers with primary education, those whose mothers had high school or university education had a higher probability of dietary supplement use (OR: 2.17, 95% CI: 1.17–4.01 and OR: 2.16, 95% CI: 1.14–4.07, respectively). In comparison to respondents who assessed a familiar socioeconomic status as very good or good, children living in families with an average socioeconomic status had significantly higher odds ratios of VMS use (OR: 1.69, 95% CI: 1.16–2.48). Respondents who spent 1 to 5 h/week on being physically active (not including gymnastic classes at school) were 1.44-fold (95% CI: 1.02–2.04) more likely to be VMS users than those being physically active <1 h/week. Moreover, overweight and obese children (BMI ≥ 25 kg/m^2^) were less likely to be supplement users (OR: 0.67, 95% CI: 0.46–0.98) than those with a normal BMI range (18.5–24.9 kg/m^2^). An inverse statistically significant trend between BMI and usage of VMSs was observed, each 1 kg/m^2^ increment in BMI was associated with a 7% (95% CI: 2–12%; *p*-trend = 0.008) lower probability of VMS use. Moreover, children who suffer from chronic diseases were more likely to be supplement users (OR: 2.32, 95% CI: 1.46–3.69) than those without chronic diseases. The prevalence of VMS usage was 3.79-fold (95% CI: 2.54–5.63) higher among those who were fortified product consumers vs. non-consumers. Children who intentionally modified their diet by including or excluding some food products had a 1.60-fold (95% CI: 1.01–2.53) higher probability of usage of VMS and those who simultaneously included and excluded food products had a 2.22-fold (95% CI: 1.29–3.81) higher probability of usage of VMS compared to non-consumers.

Compared to children, in adolescents, some different factors like gender or residential area were associated with VMS usage; however, some of them, like mother’s education level, fortified food consumption, or diet modification towards better food choices, were overlapped with those determined for children (Table 3). VMS users vs. non-users were less likely to be male (OR: 0.56, 95% CI: 0.37–0.87) and live in rural areas (OR: 0.63, 95% CI: 0.40–0.99). In comparison to adolescents whose mothers had primary education, those with university mothers’ education had a higher probability of VMS use (OR: 5.19, 95% CI: 1.72–15.6). Adolescent who were underweight (BMI < 18.5 kg/m^2^) were less likely to be supplement users (OR: 0.35, 95% CI: 0.17–0.73) than those with a normal BMI (18.5–24.9 kg/m^2^). In contrast to children, each 1 kg/m^2^ increment in BMI was associated with a non-significant 4% (95% CI: −2–10%; *p*-trend = 0.22) higher probability of VMS usage. It was found that adolescents who declared average or poor health status vs. those who declared at least a good health status were more likely to be supplement users (OR: 1.96, 95% CI: 1.13–3.39). Moreover, the prevalence of VMS use was 2.54-fold (95% CI: 1.62–4.00) higher among those who were fortified food consumers vs. non-consumers; 2.09-fold (95% CI: 1.24–3.52) higher among those who intentionally included or excluded some food products; and 3.02-fold (95% CI: 1.71–5.35) higher among those who simultaneously included and excluded some food products vs. those who not modified their diet.

## 4. Discussion

The study showed that the majority of significant predictors of VMS use were different in children and adolescents. In children, socioeconomic status, physical activity level, BMI, and presence of chronic disease were determinants of dietary supplement use; while in adolescents, it was gender, residential area, BMI (in opposite trend compared to children), and health status. Notwithstanding, some determinants such as higher mothers’ education, consumption of fortified foods, and declaration of diet modifications overlapped in both groups.

In our study, a significantly higher number of children (39.5%) compared to adolescents (20.3%) used VMSs. Similarly, in the National Health Interview Survey 2007 [2] and the National Health and Nutrition Examination Survey (NHANES) 1999–2004 [16], these products were used by more children (aged 5–11) (38.9% and 37.4%, respectively) than adolescents (12–17 years) (23.0% and 26.6%, respectively).

A higher family income was associated with a higher use of dietary supplement in studies conducted among American children and adolescent [2,10,15,16,18]. In our study, children with an average socioeconomic status compared with those with very good/good socioeconomic status had a higher probability of VMS use; however, our study was based on a self-assessment of socioeconomic status, not on exact income, which was reported in other surveys [2,10,15,18].

A higher level of parents’ education was associated with an increased probability of supplement use in both examined groups. Similar observations have been reported from studies conducted in the US children and adolescent populations [2,10]. It could be explained by the increased awareness of a healthy lifestyle in people with higher education and by the fact that supplement usage is commonly considered as a pro-healthy behaviour. In fact, dietary supplements are not routinely recommended for children and adolescents who consume a varied diet. NHANES 2003–2006 results suggested that it is a controversial strategy to improve nutrient intakes [25]. Furthermore, the results of some studies indicate that unjustified supplementation may increase the total mortality risk in the population as well as incidence of specific diseases [7,26,27]. However, for certain groups, for example, children with nutritional deficiencies (e.g., vitamin D supplementation during low sunlight exposure), malabsorption or obese children in weight loss programs, VMS use could be recommended [28].

In this study, children who spent 1–5 h per week compared to those who spent <1 h/week of free time being physically active (swimming, playing football, dancing, playing tennis or other sports) had a higher probability of VMS use. Similarly, in studies conducted among American children and adolescents [11,12,14], Finnish [5] and Slovenian [6] adolescents, supplement users vs. non-users were more frequently physically active as well as less likely to watch television or video/computer games [14,15]. In American [14,15] and Korean [17] studies, overweight and obese children and adults were less likely to be supplement users. In our study, the usage of VMSs was associated with a normal BMI; children who were overweight and obese, and adolescents who were underweight had a lower probability to be supplement users.

Adolescents who declared average or poor health status and children suffering from chronic diseases compared to healthy respondents were more likely to consume VMSs. In some studies, dietary supplement use was more common in children and adolescents who used prescribed medication and among those who suffered from chronic diseases or were complaining about health [5,17]. On the contrary, in large American studies, children and adolescents who were supplement users compared to non-users more frequently declared very good or excellent health status [2,13].

Children and adolescents who were VMS users more often consumed fortified food and modified their diet towards better food choices. It was consistent with the results of other studies conducted in children and adolescents, where supplement use was associated with more healthful food choices [11], with higher diet quality score, regular consumption of some meals, low-fat foods [12,17] as well as more healthful food patterns such as higher intakes of whole grains, fruit, vegetables, and lower intakes of soft drinks, fried food, and meat [11,12,14].

We hypothesise that the differences in the determinants of dietary supplements may be a result of motivation for using these products. Parents make this decision in order to improve the nutritional habits of children, because of poor appetite or frequently occurring colds [10]; while adolescents are buying supplements by themselves, expecting a variety of effects, such as increased energy, building muscle mass or decreased weight and enhanced physical appearance, which often reflected the extensive marketing of specific dietary supplements [29]. In our study, as the main reasons for VMS use, parents of children pointed out improving children’s overall health and a diet poor in nutrients; while adolescents declared improving overall health and physician recommendations. Similarly, in the American population, the main motivation of children and adolescents to use VMSs was improving overall health and preventing nutrient deficiencies [2,30].

The strengths of the present study include a large number of respondents as well as VMS users, and the detailed characteristics of participants by socio-demographic and lifestyle factors. It provided an opportunity to use the logistic regression models to determine factors significantly associated with VMS use separately in children and adolescents as well as allowed to show differences in the determinants of VMS usage between both groups. As in all observational studies, unmeasured or residual confounding cannot be disregarded. In the study, a limited number of determinants was examined; it is highly probable that some of the important factors of VMS use were not taken into account. Another limitation of this study was that the survey might not be representative for all primary and secondary school students from Central-Eastern Poland, since the study was carried out only in those schools in which the headmasters gave consent to it. Moreover, it is possible that respondents with a more health-oriented lifestyle are more likely to participate in the study. Furthermore, the methodology was not uniform; in children (5–12 years), the survey was completed by parents, while in adolescents (13–20 years) by the students themselves; this could have an effect on the outcomes.

## 5. Conclusions

Socio-demographic and lifestyle factors associated with VMS use may vary by age groups among school students. Children with an average socioeconomic status, who spent more free time on being physically active, with a normal body weight, and who suffered from chronic diseases, were more likely to use supplements. In adolescents, VMS users compared with non-users more often were female and lived in urban areas, less likely were underweight and assessed their health status as average or poor. In both groups, higher mothers’ education, consumption of fortified food, and declaration of diet modifications towards better food choices were predictors of VMS use. Since the age of the respondents may determine different behaviours associated with the use of dietary supplements, further research should be conducted also among other age groups. Understanding the determinants affecting the use of dietary supplements in children and adolescents may identify the risk to subgroup populations of the incorrect use of dietary supplements, as well as allow the planning of appropriate public health education; therefore, further research is warranted.

## Figures and Tables

**Table 1:** Prevalence of specific types of vitamin/mineral supplement (VMS) use and reasons for using or non-using them by children (*n* = 762) and adolescents (*n* = 816).

	Children ≤12 years	Adolescents >12 years
Parameters		
	Users ^a^	Users ^a^
	*n* = 301 %	*n* = 166 %
Type of VMS		
Single vitamin	20.2	25.9
Single mineral ^b^	4.3	16.9
Multivitamin and/or mineral(s)	39.9	33.1
Vitamin(s)/mineral(s) + other ingredient ^b^	72.1	42.8
Usage more than one VMS	13.3	12.0
Reason for using VMSs ^c^		
Improve overall health	77.4	76.5
Diet poor in nutrients ^b^	45.2	20.5
Physician recommendation ^b^	32.2	26.5
Necessary when medicines are used ^b^	10.6	4.8
Other	15.0	9.0
	Non-users	Non-users
	*n* = 461 %	*n* = 650 %
Reason for avoidance VMSs ^c^		
No need to use because of proper nutrition ^b^	62.3	44.2
Lack effect on health improvement ^b^	24.7	7.8
It can be harmful ^b^	22.3	7.2
Too high price ^b^	20.8	13.1
Other ^c^	9.1	3.4

^a^ supplement user—a person who used one or more VMSs over the 12 months before the survey; ^b^ a statistically significant difference between the group of children and adolescents; chi-square test, *p*-value < 0.05; ^c^ each respondent could select one or more answers.

**Table 2:** The distribution of respondents using specific nutrients with vitamin/mineral supplements (VMSs) stratified by duration of use (*n* = 467).

VMS Using	*n* (%) *	*p*-value	Short-Term Users 7 days–<1 month *n* = 147 % **	Medium-Term Users 1–3 months *n* = 214 % **	Long-Term Users >3 months *n* = 106 % **	*p*-value
Total ^b,c^						
children	301 (100)	0.012	29.9	43.9	26.2	0.048
adolescents	166 (100)		34.3	49.4	16.3	
Vitamin A						
children	215 (71.4)	<0.001	22.3	48.4	29.3	0.131
adolescents	50 (30.1)		14.0	64.0	22.0	
Vitamin E						
children	192 (63.8)	<0.001	26.0	47.4	26.6	0.236
adolescents	51 (30.7)		19.6	60.8	19.6	
Vitamin D						
children	217 (72.1)	<0.001	22.6	47.9	29.5	0.279
adolescents	46 (27.7)		13.0	58.7	28.3	
Vitamin C ^c^						
children	252 (83.7)	<0.001	30.5	43.3	26.2	0.049
adolescents	109 (65.7)		29.4	55.0	15.6	
Vitamin B_1_^a,c^						
children	174 (57.8)	<0.001	24.1	46.0	29.9	0.010
adolescents	40 (24.1)		12.5	72.5	15.0	
Vitamin B_2_^a,c^						
children	189 (62.8)	<0.001	27.5	44.4	28.1	0.021
adolescents	41 (24.7)		17.1	68.3	14.6	
Niacin ^a^						
children	197 (65.4)	<0.001	25.4	46.7	27.9	0.041
adolescents	41 (24.7)		14.6	68.3	17.1	
Vitamin B_6_^a,c^						
children	202 (67.1)	<0.001	26.7	46.6	26.7	0.003
adolescents	55 (33.1)		12.7	72.7	14.6	
Folic acid						
children	138 (45.8)	<0.001	19.6	50.0	30.4	0.064
adolescents	41 (24.7)		12.2	70.7	17.1	
Vitamin B_12_^a,c^						
children	167 (55.5)	<0.001	22.8	47.3	29.9	0.016
adolescents	40 (24.1)		12.5	72.5	15.0	
Biotin						
children	127 (42.2)	<0.001	18.9	52.8	28.3	0.328
adolescents	28 (16.9)		10.7	67.9	21.4	
Pantothenic acid						
children	135 (44.9)	<0.001	25.9	48.2	25.9	0.061
adolescents	39 (23.5)		12.8	69.2	18.0	
Calcium						
children	76 (25.2)	0.900	18.4	55.3	26.3	0.828
adolescents	41 (24.7)		21.9	56.2	21.9	
Magnesium						
children	27 (9.0)	<0.001	29.6	48.1	22.3	0.532
adolescents	44 (26.5)		18.2	56.8	25.0	
Iron						
children	35 (11.6)	0.010	22.9	51.4	25.7	0.616
adolescents	34 (20.5)		14.7	61.8	23.5	
Zinc ^a,c^						
children	87 (28.9)	0.210	23.0	41.4	35.6	0.015
adolescents	39 (23.5)		12.8	69.2	18.0	

*p*-value was determined using the chi-square test; different letters in superscript indicate statistically significant differences between: ^a^ short-term and medium-term users, ^b^ short-term and long-term users, ^c^ medium-term and long-term users; * the percentage of subjects using specific nutrients was given in relation to total VMS users in children (*n* = 301) and adolescents (*n* = 166); ** percentages were calculated in relation to children or adolescents who used supplements containing specific nutrients (percentages are summarized in rows).

**Table 3:** Logistic regression of vitamin/mineral supplement (VMS) use by socio-demographic and lifestyle determinants in school children and adolescents.

Study Factors	Children ≤12 years (*n* = 762)		Adolescents >12 years (*n* = 816)	
	Crude	Multivariate-Adjusted	Crude	Multivariate-Adjusted
	OR (95% CI)	OR (95% CI)	OR (95% CI)	OR (95% CI)
Age (years)	0.96 (0.87–1.06)	1.01 (0.90–1.14)	0.93 (0.86–1.01)	1.15 (0.95–1.39)
*P* for trend	0.39	0.86	0.09	0.15
Gender				
Female	1.00	1.00	1.00	1.00
Male	0.96 (0.72–1.28)	0.94 (0.68–1.30)	0.75 (0.51–1.09)	**0.56 (0.37–0.87)**
Residential area				
Urban	1.00	1.00	1.00	1.00
Rural	1.04 (0.70–1.57)	1.43 (0.88–2.35)	0.94 (0.67–1.33)	**0.63 (0.40–0.99)**
Mother’s education level				
Primary	1.00	1.00	1.00	1.00
High school	1.87 (1.10–3.18)	**2.17 (1.17–4.01)**	2.10 (0.92–4.78)	2.02 (0.85–4.79)
University	2.05 (1.23–3.40)	**2.16 (1.14–4.07)**	6.07 (2.20–16.8)	**5.19 (1.72–15.6)**
Socioeconomic status				
Very good or good	1.00	1.00	1.00	1.00
Average	1.38 (0.99–1.91)	**1.69 (1.16–2.48)**	0.76 (0.52–1.11)	0.75 (0.49–1.15)
Poor	0.64 (0.32–1.32)	1.12 (0.49–2.54)	0.59 (0.33–1.04)	0.83 (0.44–1.56)
Physical activity (h/week)				
<1	1.00	1.00	1.00	1.00
1–5	1.72 (1.26–2.36)	**1.44 (1.02–2.04)**	1.52 (1.07–2.17)	1.26 (0.85–1.86)
≥6	1.35 (0.76–2.41)	1.21 (0.63–2.32)	1.10 (0.49–2.48)	0.87 (0.36–2.13)
Body Mass Index (kg/m^2^)				
<18.5	0.99 (0.64–1.52)	1.10 (0.69–1.76)	0.39 (0.20–0.77)	**0.35 (0.17–0.73)**
18.5–24.9	1.00	1.00	1.00	1.00
≥25	0.62 (0.44–0.88)	**0.67 (0.46–0.98)**	1.14 (0.71–1.84)	0.99 (0.58–1.70)
*P* for trend	0.003	0.008	0.19	0.22
Health status				
At least good	1.00	1.00	1.00	1.00
Average or poor	0.70 (0.37–1.32)	0.56 (0.27–1.17)	1.41 (0.89–2.24)	**1.96 (1.13–3.39)**
Current chronic diseases				
No	1.00	1.00	1.00	1.00
Yes	2.24 (1.52–3.31)	**2.32 (1.46–3.69)**	1.90 (0.99–3.68)	1.27 (0.61–2.66)
Special diet				
No	1.00	1.00	1.00	1.00
Yes	1.58 (0.88–2.83)	0.82 (0.40–1.66)	1.57 (0.96–2.58)	1.02 (0.56–1.85)
Number of meals/day				
≤3	1.00	1.00	1.00	1.00
4	1.38 (0.86–2.20)	1.16 (0.69–1.96)	1.52 (1.04–2.22)	1.46 (0.95–2.25)
≥5	1.82 (1.09–3.01)	1.68 (0.96–2.95)	1.47 (0.90–2.41)	1.51 (0.86–2.65)
Fortified food consumption				
No	1.00	1.00	1.00	1.00
Yes	3.39 (2.35–4.89)	**3.79 (2.54–5.63)**	3.12 (2.04–4.77)	**2.54 (1.62–4.00)**
Diet modification				
Lack of modification	1.00	1.00	1.00	1.00
Excluding or including some foods	1.76 (1.17–2.64)	**1.60 (1.01–2.53)**	2.36 (1.51–3.70)	**2.09 (1.24–3.52)**
Simultaneously excluding and including some foods	2.95 (1.83–4.75)	**2.22 (1.29–3.81)**	3.00 (1.85–4.87)	**3.02 (1.71–5.35)**

Supplement user—a person who used one or more VMSs over the 12 months before the survey. Notes: bold font indicates the statistical significant results in the multivariate-adjusted analysis.

## Supplementary Materials

The following are available online at https://www.mdpi.com/2072-6643/11/3/658/s1. Table S1. Characteristics of children (*n* = 762) and adolescents (*n* = 816) by socio-demographic and lifestyle determinants stratified by vitamin/mineral supplement (VMS) use.
