# Supplementary material for: Different Socio-Demographic and Lifestyle Factors Can Determine the Dietary Supplement Use in Children and Adolescents in Central-Eastern Poland
Source: Nutrients. 2019 Mar 18;11(3):658. doi: 10.3390/nu11030658 (PMC6470763; doi:10.3390/nu11030658)
Supplement: Supplementary file 1 [file nutrients-11-00658-s001.pdf]

Supplementary Material Table S1. Characteristics of children (n=762) and adolescents (n=816) by socio-demographic and lifestyle determinants stratified by vitamin/mineral supplement (VMS) use

Parameters	Children ≤12 years		*P*-value [c]	Adolescents >12 years		*P*-value [c]
	Users [a] n = 301 %	Non-users n = 461 %		Users [a] n = 166 %	Non-users n = 650 %	
Gender						
Female	50.5	49.5	0.779	71.7	65.4	0.124
Male	49.5	50.5		28.3	34.6	
Residential area [b]						
Urban	84.6	85.1	0.835	43.9	42.4	0.726
Rural	15.4	14.9		56.1	57.6	
Mother's education level [b]						
Primary	8.1	14.8	0.019	16.7	34.9	0.001
High school	34.9	34.1		55.6	55.5	
University	57.0	51.1		27.7	9.6	
Socioeconomic status [b]						
Very good or good	65.1	69.6	0.053	61.5	52.9	0.108
Average	31.2	24.3		28.9	32.9	
Poor	3.7	6.1		9.6	14.2	
Physical activity (h/week) [b, c]						
< 1	31.2	43.2	0.003	41.6	51.2	0.060
1-5	61.2	49.0		53.6	43.4	
≥ 6	7.6	7.8		4.8	5.4	
Body Mass Index (kg/m^2) [b]						
< 18.5	15.1	13.4	0.019	6.0	14.4	0.014
18.5 – 24.9	61.3	53.5		77.6	72.3	
≥ 25	23.6	33.1		16.4	13.3	
Health status [b]						
At least good	95.3	93.4	0.281	83.1	87.9	0.114
Average or poor	4.7	6.6		16.9	12.1	
Current chronic diseases						
No	77.1	88.3	<0.001	91.6	95.4	0.052
Yes	22.9	11.7		8.4	4.6	
Special diet						
No	92.0	94.8	0.124	84.9	89.9	0.073
Yes	8.0	5.2		15.1	10.1	
Number of meals/day						
≤ 3	10.0	14.3	0.052	33.1	43.4	0.057
4	57.1	59.7		47.6	40.3	
≥ 5	32.9	26.0		19.3	16.3	
Fortified food consumption						
No	15.3	38.0	<0.001	18.1	40.8	<0.001
Yes	84.7	62.0		81.9	59.2	
Diet modification						
Lack of modification	65.1	80.3	<0.001	59.0	79.1	<0.001
Excluding or including some foods	18.3	12.8		21.7	12.3	
Simultaneously excluding and including some foods	16.6	6.9		19.3	8.6	

[a] supplement user – the person who used at least one VMS within at least one week or longer over the past 12 months

[b] missing data: place of residential area (1.4%), educational level of mother (39.7%), socioeconomic status of family (6.5%), BMI (1.4%) and health status (2.5%)

[c] time spent on being physically active, not including gymnastic classes at school

[d] chi-square test
